# Lower serum 25-hydroxycholecalciferol is associated with depressive symptoms in older adults in Southern Brazil

**DOI:** 10.1186/s12937-020-00638-5

**Published:** 2020-11-14

**Authors:** Gilciane Ceolin, Luísa Harumi Matsuo, Susana Cararo Confortin, Eleonora D’Orsi, Débora Kurrle Rieger, Júlia Dubois Moreira

**Affiliations:** 1 Postgraduate Program in Nutrition, Federal University of Santa Catarina, Florianopolis, Brazil; 2 Postgraduate Program in Public Health, Federal University of Maranhão, Maranhão, Brazil; 3 Department of Public Health, Postgraduate Program in Public Health, Federal University of Santa Catarina, Florianopolis, Brazil; 4 Department of Nutrition, Translational Nutritional Neuroscience Working Group, Postgraduate Program in Nutrition, Federal University of Santa Catarina, Florianopolis, Brazil; 5 Translational Nutritional Neuroscience Working Group. Postgraduate Program in Nutrition. Department of Nutrition, Universidade Federal de Santa Catarina. Centro de Ciências da Saúde, Campus Universitário – Trindade, Florianópolis, Santa Catarina 88040-900 Brazil

**Keywords:** Vitamin D, Aging, Depressive symptoms, cohort study, Mental health

## Abstract

**Background:**

Older adults are one of the most susceptible populations to depression, especially those living in low- and middle-income countries. As well, they are also considering a risk group for vitamin D deficiency. Low serum vitamin D has been associated with an increased risk of brain neuropsychiatry disorders. We aimed to investigate the association between serum 25-hydroxycholecalciferol concentrations and depressive symptoms in adults aged 60 years and over from southern Brazil.

**Methods:**

A cross-sectional analysis was performed using data collected during 2013–2014 from the populational-based longitudinal EpiFloripa Aging Study (*n* = 1197). Serum 25-hydroxycholecalciferol concentrations were analyzed and classified according to the Endocrine Society reference values [sufficiency (≥ 30 ng/mL), insufficiency (21–29 ng/mL), and deficiency (≤ 20 ng/mL)]. Depressive symptoms were evaluated using the Geriatric Depression Scale (15-item GDS). Logistic regression was performed to assess depressive symptoms in each vitamin D category. The analysis was adjusted for sex, age, skin color, family income, leisure-time physical activities, social or religious groups attendance, morbidities, cognitive impairment, and dependence in activities of daily living.

**Results:**

A total of 557 participants with complete data for exposure and outcome were enrolled in the analysis. Most of the sample participants were female (63.1%), age-range 60–69 years (42.2%), white skin color (85.1%), and vitamin D serum level samples were collected in autumn (50.7%). Depressive symptoms were present in 15.8% of the participants, and the prevalence was higher in individuals classified as deficient in vitamin D (23.2, 95% confidence interval [CI] = 15.6;32.9) and insufficiency (17.2, 95%CI = 11.0;25.9). The crude analysis showed that vitamin D deficient participants had 3.08 (CI = 1.53;6.20) times higher odds to present depressive symptoms compared to vitamin D sufficiency. After adjusting, the association was maintained [OR 2.27 (95%CI = 1.05;4.94).

**Conclusions:**

Serum 25-hydroxycholecalciferol deficiency was positively associated with depressive symptoms in older adults from southern Brazil.

## Introduction

Despite efforts to reduce the number of people with depressive disorders, their prevalence has increased, particularly in lower-income countries, and depression holds the third position globally in terms of years lived with disability [1, 2]. Besides being a debilitating disease for older adults, presenting a variety of emotional and physical problems, its treatment approach involves mainly implementation of preventive health habits and high-cost rehabilitation programs [3, 4].

Depression in older adults living in low- and middle-income countries is associated with a higher risk of suicide and excess mortality, more frequent medical consultations and hospitalization, and a significant family burden, even though the number of depressive older adults is similar to that of adults [3, 5, 6]. Additionally, depression is associated with significant adverse consequences ranging from poor quality of life, difficulties with daily living activities (DLA), physical comorbidities, and cognitive impairment [5]. Environmental causes and lifestyle factors, such as obesity, exposure to physical and substance abuse, widowhood, the presence of chronic illnesses and sleep disorders, lack of education and social support, intimate partner, and physical activity have been related to depression [7]. Several mechanisms have been studied in the neurobiology of depression, such as genetic factors, neurotransmitter systems, neuroendocrine systems, inflammation, functional and structural brain anatomy, and cognition [8, 9]. Some promising studies suggest the involvement of nutritional factors, such as vitamin D, in the development of depressive symptoms [10, 11].

Vitamin D-related effects on homeostasis, neurotrophic, and neuroimmunomodulatory actions appear to be associated to depressive symptoms prevention. However, the exact molecular mechanisms underlying this relationship are not well stablished [12]. Vitamin D is considered a neurosteroid hormone because of its essential role in the central nervous system and connection to processes associated with cell differentiation, neurotrophic factor synthesis and release, neurotransmitter synthesis, intracellular calcium homeostasis, redox balance, neuronal metabolism, and cognitive function [13, 14]. Vitamin D signaling through the nuclear receptors vitamin D receptors (VDR) and the membrane receptor protein disulfide isomerase family member3 (PDIA3), and the presence of some key enzymes of Cytochrome P450, such as CYP27a1, CYP27b1 and CYP24a1, responsible to convert the inactive form of vitamin D to its active form in the brain cells, as well as its requirements for neuron cell cycle, initiated the investigation of vitamin D relevance for brain metabolism in health and disease [15–18]. One underlying mechanism proposed is that the vitamin D could regulate serotonin synthesis [19, 20]. Calcitriol (1,25-dihydroxyvitamin D3) is a key regulator of serotonin, inducing the TPH2 (tryptophan hydroxylase 2) gene expression, which is the enzyme involved on tryptophan metabolism in the brain to produce serotonin [19, 21]. Calcitriol also reduce serotonin reuptake transporter (SERT) expression, which is responsible to remove serotonin from synaptic cleft, and monoamine oxidase-A (MAO-A), which is responsible for serotonin catabolism [22]. Calcium homeostasis is another possible mechanism associated to vitamin D neuroprotection. Its relevance in redox balance and inflammation could also be involved in the relationship between vitamin D and depression [23, 24]. Vitamin D stimulates the expression of many antioxidant genes, such as factor 2 related to the nuclear factor eritroid-2 (NRF2), g-glutamyl transpeptidase (g-GT), glutamate-cysteine ligase (GCLC), glutathione reductase (GR), glutathione peroxidase (Gpx) [23].

Aging process is associated to the reduced ability to sustain homeostasis and more susceptibility to pathological alterations, such as neuropsychiatric disorders [25, 26]. Understanding the link between vitamin D concentration and depression in aging is required due to be a possible way to minimize the effects of depression. Given the complexity of the condition, more risk to mortality, difficulty in access to treatment, and including that older adults that are undiagnosed [6, 27, 28]. Besides of being considered a risk group for depression, older adults are also one of the risk groups for vitamin D deficiency due to their reduced skin capacity to synthesize vitamin D, reduced sun exposure, and more significant complications related to low serum vitamin D concentrations [29, 30]. Some observational studies have investigated the relationship between low serum vitamin D concentrations and depressive symptoms in adulthood or in adults and elderly population combined, and few included only older adults in the sample, as observed by a recently published meta-analyses [31–33].

Therefore, the present study aimed to investigate the association between low serum vitamin D concentrations and depressive symptoms in older adults living in Southern Brazil, as well as elucidate the prevalence of depressive symptoms in this population and the magnitude of the prevalence of vitamin D deficiency as well. We hypothesized that the low serum of 25(OH) D (< 20 ng/ml) could be associated with depressive symptoms and we intend to discuss the factors related this association in elderly.

## Methods

### Study population and design

**Fig. 1:**
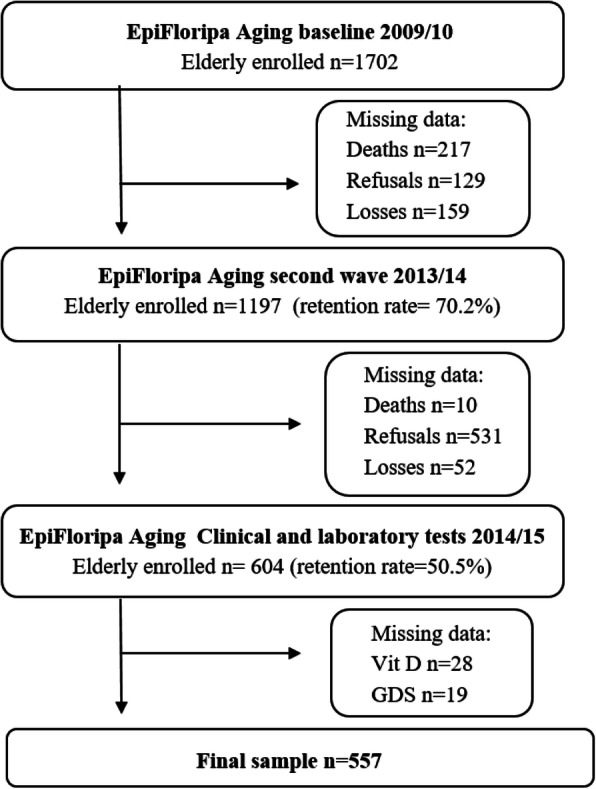
EpiFloripa Aging Study Flowchart. (GDS: Geriatric Depression Scale)

We performed a cross-sectional analysis with data collected in the second wave (2013–2014) of the populational-based EpiFloripa Aging Study. Details about the sample and methodology have been previously published [34, 35]. Briefly, EpiFloripa Aging is a cohort study aiming to investigate health determinants and aspects of the older adult population living in Southern Brazil. Older adults of both sexes, aged 60 years or over at the time of the first interview, living in the sectors determined by the survey, were considered eligible at baseline, in 2009 (Fig. 1). Older adults who were institutionalized (residing in long-term care institutions, hospitals, prisons) were excluded.

All the older adults in the follow-up (1197 interviews; retention rate of 70.2%) were invited to participate in the second wave of the study (2013–2015), which included biochemical blood analysis (retention rate of 50.5%; *n* = 604) [35]. For this cross-sectional analysis, we enrolled 577 individuals, and the inclusion criteria were to present complete data for depressive symptoms and to be the evaluation of 25-hydroxycholecalciferol (Fig. 1).

The EpiFloripa Aging Study’s second wave of follow-up was approved by the Human Research Ethics Committee of the Federal University of Santa Catarina (approval protocols numbers: 329.650 and 526.126). Voluntary participation of the individuals was obtained through the signing of the Informed Consent Form, after explaining the study objectives and collection procedures. The EpiFloripa Aging Study was conducted in accordance with the Declaration of Helsinki.

### Data collection and covariates

Trained interviewers conducted data collection at the participant’s home. The face-to-face interviews were scheduled with the participants by phone. EpiFloripa Aging Study data were collected using a 15-block questionnaire with socioeconomic, demographic, and health aspects related to aging and prioritizing validated instruments translated and validated in Brazil for the composition of the questionnaire to guarantee the data quality control. The selected interviewers were health science graduates with research experience. Data were recorded in a netbook and transmitted directly to the EpiFloripa database. Quality control was carried out by telephone, using a short version of the questionnaire in 10% of the samples.

### Depressive symptoms

To evaluate the presence of depressive symptoms, we applied the 15-item Geriatric Depression Scale (15-item GDS). We used the cut-off points suggested by Almeida and Almeida, we classified participants into two groups: those with the presence of depressive symptoms (≥ 6 points) and those with the absence of depressive symptoms (≤ 5 points) [36, 37].

The GDS-15 is recommended by the Brazilian Ministry of Health, and is a useful alternative for the rapid assessment of the presence of depressive symptoms in the elderly [38]. The GDS-15 was translated and validated in Brazil according to ICD-10 criteria for research and DSM-IV with outpatients aged 60 or over who met criteria for depressive disorder (current or in remission) [36, 39]. The cutoff point 5/6 (not case/case) produced sensitivity indexes of 85.4% and specificity of 73.9% according to ICD-10 and 90.9% of sensitivity and 64.5% of specificity according to the DSM-IV and the internal consistency using Cronbach’s alpha coefficient revealed reliability indexes of 0.81 [39]. Moreover, in the test-retest reliability when the outpatient was evaluated twice in 48 to 72 h, and GDS-15 scores were reasonably stable, as assessed by paired Wilcoxon (z = 1.60, *p* = 0.109) and Spearman’s correlation coefficient (rho = 0.86, *p* < 0,001) and weighted Kappa (Kappa = 0.64) [36].

### Serum vitamin D (25-hydroxycholecalciferol) concentrations

For the measurement of 25-hydroxycholecalciferol [25(OH)D] blood samples were collected at the University Laboratory for Metabolism and Dietetics, between 7 and 10 a.m., with the required minimum of 8 h fasting period before the procedure [35]. Blood samples were centrifuge (3.500 rpm) for 10 min. Serum samples used to detect 25(OH) D were immediately processed using LIASON® 25 OH vitamin D assay (Diasorin, São Paulo, Brazil) accordingly to manufacture (Functional Sensitivity: ≤ 2.0 ng/mL; (inter-assay imprecision < 20%), which is considered s a rapid, accurate, and precise assay [40]. Briefly, an antibody specific to vitamin D was coated on magnetic particles, and 25-OHD conjugated to an isoluminol derivative and diluted in phosphate buffer (pH 7.4). In the first incubation period, 25-OHD dissociated from the binding protein, and it interacts with the antibody. After the second incubation with the tracer reagent, microplate is washed with the buffer and starter reagents are added to generate the chemiluminescent signal, which is measured by a photomultiplier. Serum 25(OH) D concentrations were measured using the Microparticle Chemiluminescence method/LIAISON [41]. Subsequently, we categorized serum 25(OH) D concentrations according to the Endocrine Society Reference values [42] into: sufficiency (≥30 ng/mL), insufficiency (21–29 ng/mL), and deficiency (≤20 ng/mL).

### Covariates

For characterization of the samples, we analyzed sociodemographic variables, such as sex (male/female), skin color (white/not white), the season during blood collection (summer/autumn/winter/spring), age range (aged 60–69/70–79/≥80 years), education (no formal education/1–4/5–8/9–11/≥12 years), per capita family income in minimal wages according to the values in 2013 (R$ 678.00) and 2014 (R$ 724.00) (≤1/> 1 to ≤3/> 3 to ≤5/> 5 to ≤10/> 10), retired (no/yes), living arrangements (live with another/alone) and marital status (married/single/divorced/widowed). Behavioral modified factors were included in the analysis, such as belonging to a social or religious group (no/yes), smoking (no/yes), alcohol consumption (no/yes) collected by Alcohol Use Disorder Identification Test [43]. Leisure-time physical activity (insufficiently active < 150 min or sufficiently active ≥150 min) collected by the International Physical Activity Questionnaire [44, 45].

Health status-related information included dependence in the activities of daily living (ADLs) [no disability, low disability (any level of disability in 1 to 3 activities), and moderate/severe disability (any level of disability in ≥4 activities)] collected by the scale of daily basic and instrumental activities [46]. Screening for cognitive impairment was evaluated using the Mini-Mental State Examination (absence/presence–considering schooling, using the cut-off points 19/20 for illiterate and 23/24 for any level of education) [47, 48]. The number of comorbidities were assessed [zero/1/≥2 (sum of diagnosed diseases: spine or back pain, arthritis, cancer, diabetes, bronchitis, cardiovascular or renal conditions, tuberculosis, cirrhosis, stroke, osteoporosis, hypertension, and depression)]. Nutritional status was assessed by body mass index (underweight < 22 kg/m^2^; healthy weight 22–27 kg/m^2^; overweight > 27 kg/m^2^), according to the Nutrition Screening Initiative [49]. Information about antidepressant drug use and vitamin D supplement use was collected by consulting all boxes of medicines prescribed for and used by the participant. The Anatomical Therapeutic Chemical Classification codes from the World Health Organization Collaborating Centre for Drug Statistic Methodology were applied for codification in the database [50].

### Statistical analysis

Descriptive analysis of the data was presented as absolute and relative frequency, prevalence, and respective 95% confidence intervals (CI) for the total sample based on the presence of depressive symptoms. The distribution of the covariates was determined using the chi-square test. We performed Student’s t-test to verify the mean differences between sex and depressive symptoms in serum 25(HO) D concentrations.

Logistic regression was used to evaluate the odds ratio (OR) for depressive symptoms in each 25(HO) D category and the respective CI in the crude and adjusted association models. The covariates were included following a hierarchical model (first socioeconomic covariates, followed by behavioral covariates, and finally, health covariates). Model 1 was adjusted for demographic and socioeconomic variables (sex, skin color, age range, and family income). Model 2 included demographic, socioeconomic, and behavioral variables (leisure-time physical activities and social or religious groups attendance). Model 3 included demographic, socioeconomic, behavioral, and health variables (morbidities, cognitive impairment, dependence in activities of daily living).

The analysis was performed using Stata 14.0 software (StataCorp, College Station, TX, USA). Due to the sampling design (two-stage cluster) of the EpiFloripa Aging Study, sample weights were used in all the analyses, using the “svy” command. *P*-value < 0.05 was used to define statistical significance for all reports.

## Results

Of the 604 older adults who participated in clinical and laboratory tests in 2014–2015, 577 individuals met the criteria and presented complete data to be included in the analysis (Fig. 1). Sample characteristics are summarized according to the prevalence of depressive symptoms in Table 1. For the total sample, the prevalence of depressive symptoms was 15.8%, and the prevalence of 25(OH) D was 39.4 and 25.5% in insufficiency and deficiency cases, respectively.

**Table 1:** Prevalence of depressive symptoms according to demographic, socioeconomic, behavioral, and health characteristics

Characteristics (*n* = 557)	Total		Presence of depressive symptoms		
	n	%	%	95%CI	*P*-value
**Sex**					0.006
Men	194	36.9	11.7	6.4;20.6	
Women	363	63.1	18.2	13.5;24.0	
**Skin Color**					0.077
White	468	85.1	14.1	9.9;19.7	
Not white	89	14.9	25.5	16.6;37.0	
**Season (Vit. D collection)**					0.791
Summer	55	9.1	14.4	6.7;28.0	
Autumn	276	50.7	18.1	11.4;27.5	
Winter	145	23.6	14.8	10.2;21.0	
Spring	81	16.6	11.1	5.1;22.5	
**Age range (years)**					0.009*
60–69	236	42.2	12.4	7.2;20.5	
70–79	235	41.8	16.8	11.6;23.8	
≥ 80	86	16,0	22.2	14.3;32.9	
**Education (years)**					0.016
No formal education	35	5.5	17.5	8.4;32.9	
1–4	199	33.7	19.5	13.3;27.5	
5–8	100	19.2	14.9	8.4;25.2	
9–11	86	17.8	18.5	7.8;38.0	
≥ 12	137	23.8	8.9	4.3;17.7	
**Per capita family income (*n*** **= 539)**					0.002*
≤ 1 mw	40	6.4	22.4	11.8;38.6	
> 1 and ≤ 3 mw	156	30.3	20.6	13.7;29.7	
> 3 and ≤ 5 mw	108	17.5	19.3	11.1;31.3	
> 5 and ≤ 10 mw	136	26.4	10.9	5.6;20.1	
> 10 mw	99	19.4	9.9	4.8;19.2	
**Retirement (*n*** **= 521)**					0.152
No	102	19.9	21.8	11.5;37.1	
Yes	419	80.1	14.4	10.8;19.0	
**Living arrangements**					0.547
Live with another/others	445	78.7	16.0	12.0;21.0	
Live alone	112	21.3	15.2	8.3;26.1	
**Marital Status**					0.095
Married	317	56.5	11.6	7.7;17.2	
Single	33	6.1	20.9	7.0;48.3	
Divorced	45	9.3	27.1	14.5;44.8	
Widowed	162	28.1	19.4	13.4;17.3	
**Social and religious groups attendance**					0.114
No	298	56.3	18.2	13.1;24.8	
Yes	259	43.7	12.7	8.6;18.4	
**Alcohol Consumption**					< 0.001
No	319	56.0	19.0	13.8;25.7	
Yes	238	44.0	11.7	6.7;19.6	
**Smoking**					0.307
No	351	58.6	17.0	12.2;23.3	
Yes	206	41.4	14.1	10.1;19.3	
**Leisure-time physical activity (*n*** **= 556)**					< 0.001
Insufficiently active	395	70.7	19.3	14.7;24.8	
Sufficiently active	161	29.3	7.4	3.5;14.8	
**Dependence in ADLs (*n*** **= 554)**					< 0.001*
None	202	38.1	6.4	3.2;12.4	
Low	217	37.5	15.1	9.7;22.7	
Moderate/severe	135	24.4	30.8	21.4;42.1	
**Cognitive impairment (*n*** **= 554)**					< 0.001
Absent	442	79.6	10.9	7.3;15.9	
Present	112	20.4	34.0	23.6;46.1	
**Number of comorbidities**					< 0.001
Zero	34	6,0	13.5	2.8;46.2	
1	96	18.6	1.4	0.3;5.7	
≥ 2	427	75.4	19.5	15.1;24.9	
**Nutritional status BMI (*n*** **= 554)**					0.061
Underweight	45	8.2	23.3	12.3;39.7	
Healthy Weight	256	44.3	11.8	7.7;17.8	
Overweight	253	47.5	18.0	12.6;25.1	
**Antidepressant use (*n*** **= 529)**					0.048
No	449	85.4	15.4	11.2;20.7	
Yes	80	14.6	21.4	12.4;34.4	
**Vitamin D supplement use (*n*=521)**					0.232
No	464	90.6	16.6	12.1;22.2	
Yes	57	9.4	11.5	4.8;25.1	
**Serum 25(OH)D**					0.006*
Sufficiency	188	35.1	8.9	4.8;15.9	
Insufficiency	226	39.4	17.2	11.0;25.9	
Deficiency	143	25.5	23.2	15.6;32.9	
**Depressive Symptoms**					
Absent	467	84.2	–		
Present	90	15.8	–		

*mw* minimum wage, *ADLs* activities of daily living, *BMI* Body Mass Index, *CI* confidence interval; *P*-value, statistical significance; * *P*-value of chi-square test for trend

Individuals with presence of depressive symptoms were mostly women (18.2% *P* = 0.006), aged ≥80 years (22.2%; *P* = 0.009), with ≤4 years of formal education (19.5;17.5%; *P* = 0.016), lower per capita family income ≤1 minimum wage (22.4%; *P* = 0.002), insufficient leisure-time physical activity (19.3%; *P* < 0.001), more dependency in ADLs (30.8%; *P* < 0.001), presence of cognitive impairment (34.0%; *P* < 0.001); and reported 2 or more comorbidities (19.5% *P* < 0.001). Considering the Endocrinology Society reference values for 25(OH) D, a higher prevalence of depressive symptoms was observed in those with vitamin D deficiency than in those with sufficiency (23.8% vs. 8.9% respectively; *P* = 0.006).

On average, women presented lower serum 25(OH) D concentrations than men (*P* = 0.001), and individuals with depressive symptoms also showed lower concentrations (*P* = 0.005) (Table 2).

**Table 2:** Serum 25(OH) D concentrations according to sex and depressive symptoms

	Serum 25(OH) D (ng/mL)		
	Mean	95%CI	*P-value*
**Sex**			< 0.001
Men	28.7	27.3;30.2	
Women	25.4	24.5;26.2	
**Depressive Symptom**			0.005
Absent	27.0	26.2;27.8	
Present	24.1	22.2;26.0	

*CI* Confidence interval; *P*-value, statistical significance using Student’s T-Test

In Table 3, we present the crude and adjusted association analysis between serum 25(OH) D concentrations and depressive symptoms. In the crude analysis, we found that vitamin D-deficient individuals presented 3.08 times higher OR (CI 95% = 1.53; 6.20) of depressive symptoms than those with sufficient concentrations. After adjusting for demographic, socioeconomic, behavioral, and health covariates (model 3), vitamin D-deficient individuals presented 2.27 times higher OR (CI 95% = 1.05; 4.94) of depressive symptoms than those considered sufficient.

**Table 3:** Crude and adjusted measures of association between serum 25(OH) D concentrations and depressive symptoms

Serum 25(OH)D^1^	Crude Analysis			Model 1^**2**^			Model 2^**3**^			Model 3 (final)^**4**^		
	OR	95%CI	p	OR	95%CI	p	OR	95%CI	p	OR	95%CI	p
Sufficiency	1						1			1		
Insufficiency	2.12	0.95;4.74	0.067	1.86	0.77;4.52	0.166	1.78	0.71;4.50	0.217	2.08	0.78;5.50	0.138
Deficiency	3.08	1.53;6.20	0.002	2.82	1.37;5.81	0.005	2.61	1.22;5.60	0.014	2.27	1.05;4.94	0.038

^1^References by the Endocrine Society; ^2^Model 1 was adjusted by demographic and socioeconomic factors (sex, skin color, age range, family income); ^3^Model 2 was adjusted by demographic, socioeconomic, and behavioral factors (leisure-time physical activities and social or religious groups attendance); ^4^Model 3 was adjusted by demographic, socioeconomic, behavioral, and health factors (morbidities, cognitive impairment, dependence in activities of daily living). OR, odds ratio; CI, confidence interval

## Discussion

In the present study, we observed a statistically significant association between serum 25(OH) D deficiency and depressive symptoms in the older adult population living in one capital of southern Brazil, even after adjustments for potential confounding factors related to demographic, socioeconomic, behavioral, and health variables. Vitamin D deficient individuals had 2.7 times higher odds of depressive symptoms. We also found that 15.8% of the population enrolled in the analysis presented depressive symptoms, using the 15-item GDS; thus, showing the importance of screening for depression symptoms in this population.

There is little evidence published in the literature about vitamin D deficiency in older adults and its relationship with neuropsychiatric disorders, especially studies that used a population-based sample, as presented here. Until May 2020, we found 10 other studies with cross-sectional analysis performed with older adults [51–60], one of these with longevous individuals (aged > 100 years) [42], and two were conducted only with men [57, 58]. *We identified* 13 cross-sectional studies conducted with a mixed population of adults and older adults [61–73]. Only two studies were performed in low- and middle-income countries [61, 72], which are the most affected by depression accordingly to World Health Organization [1]. We concluded that there is a gap in the literature about investigations involving depressive symptoms in low- and middle-income countries. This was a relevant aspect because socioeconomic covariates, such as low per capita family income and fewer years of formal education, were related to a higher prevalence of depressive symptoms in our sample, and were a universal life situation for the populations living in low- and middle-income countries [74, 75].

Older adults are considered a risk group for hypovitaminosis D due to the reduction in vitamin D absorption and synthesis, modification in food intake that could reduce vitamin D ingestion, and reduced outdoor activity that favors sunlight-derived vitamin D synthesis [29, 76–78]. A study comparing two cohorts from the Longitudinal Aging Study Amsterdam (LASA), one with adults aged 55–65 years (*n* = 737) and other with older adults aged 65 years or older (*n* = 1282), found a higher 25(OH) D concentrations in the younger group, better physical functioning, fewer chronic diseases, and they were more physically active compared with the older ones [73].

In our sample, 70.7% of the participants who reported reduced leisure physical activity and 19% of those who presented with depressive symptoms were classified as vitamin D insufficient (data not shown). These data were consistent with the literature review. A recent meta-analysis showed that people with depression were less physically active than the corresponding controls, and about 80% of people with depression were unable to achieve the recommended weekly physical activity [79]. A previous study found that less physical activity was associated with reduced vitamin D concentrations and modestly attenuated the OR for depression in the adjusted analysis [52]. Another study provided evidence for the potential mediating role of physical functioning in the relationship between low 25(OH) D levels and increasing of depressive symptoms [73].

We found a relationship between sex and the mean 25(OH) D concentration. Women presented lower levels than men, but we could not perform a stratified analysis by sex due to the small number of observations in the variable categories for men. However, we included sex as an adjustment variable in the investigation. Previous studies described sex differences showing generally lower 25(OH) D concentrations in women than in men [51–53, 55, 73, 80]. The sex difference is not yet clearly understood, but is considered to be related to sunscreen use and body fat mass [78, 81, 82]. In our study (data not shown), overweight was found to have a higher prevalence in women (68.8% vs. 31.2% women and men, respectively; *P* = 0.012) and those with hypovitaminosis D (43.9, 29.6 and 26.5% in insufficiency, deficiency, *and* sufficiency, respectively, *P* = 0.001).

In our study, we observed a prevalence of ~ 15% for depressive symptoms, which was considered a high prevalence [1]. Other studies that evaluated depressive symptoms using the GDS screening tool, also showed higher prevalence: 27.9% in South Korea (sample included individuals aged ≥65 years; *n* = 2853) [53]; 25.2% in England (sample included those aged ≥65 years; *n* = 2070) [54], and 32.2% in China (*n* = 940) [56]. One study performed in the Netherlands found 7% prevalence (sample included individuals aged ≥65 years; *n* = 2839) [59]. In our study, women presented a higher prevalence (18%) of depressive symptoms, which could indicate this group’s greater vulnerability for depression. The studies mentioned above also showed a higher prevalence 30.9% vs. 21,9% [53], 35.6% vs. 17.7% [56], and 40,6% vs. 23.8% in women vs. men, respectively, the last one being an Italian sample (individuals aged ≥65 years; *n* = 1.675) [40].

The prevalence of depressive symptoms presented an inverse trend in 25(OH) D concentrations with 8.9% of individuals with levels ≥30 ng/mL, 17.2% with 21–29 ng/mL, and 23.2% with ≤20 ng/mL. Our findings were lower than those of a previous study. We determined 22.6% with levels < 30 ng/mL, 25.8% with < 20 ng/mL, and 35.0% with < 10 ng/mL levels of serum concentration [54]; however, they followed the same trend. In general, studies demonstrated that a lower serum vitamin D level was associated with a higher prevalence of depressive symptoms, even when other reference values were used [6% with > 71.7 nmol/L; 4.6% with 53.4–71.7 nmol/L; 5% with 36.7–53 nmol/L; and 11% with < 36.7 nmol/L [59]; as well as 7.8% with ≥30.0 ng/mL; 27.4% with 20.0–29.9 ng/mL; 50.1% with 10.0–19.9 ng/mL; and 14.7% with < 10.0 ng/mL [53]. The higher chance of depressive symptoms in older adults with low 25(OH) D concentrations was consistent with previous cross-sectional studies’ results [51, 54, 56, 59, 60]. In Amsterdam, the Netherlands, in a population-based sample (≥65 years; *n* = 1282), the severity of depressive symptoms evaluated using the Center for Epidemiologic Studies Depression Scale (CES-D) was associated with decreased serum 25(OH) D concentrations (B = 9.6; 95%CI = 16.9;2.4; *p* = 0.01) [51]. In England, in a Health Survey with older adults (≥65 years; *n* = 2070), depressive symptoms evaluated by 10-item GDS were associated with clinical vitamin D deficiency (25(OH) D levels < 10 ng/mL (OR = 1.46; 95%CI = 1.02;2.08; *p* = 0.04) [54]. Data from the English Longitudinal Study of Aging (≥50 years; *n* = 5870) showed a significant association between low 25(OH) D concentrations and depressive symptoms (CES-D) [OR = 1.58; 95% CI = 1.20–2.07 for the lowest quartile; OR = 1.45, 95%CI = 1.15.1.83 for ≤30- nmol/L cut-off and OR = 1.34, 95%CI = 1.10–1.62 for the ≤50 nmol/L cut-off)] [63].

Although depression has well-studied pathophysiology, the biochemical mechanisms involved in the relationship between vitamin D and depression are still not well elucidated. Some mechanisms of vitamin D have been suggested, such as changes in glutamatergic neurotransmitter and monoaminergic systems, interactions with inflammatory processes, and control of the expression of those genes that are responsible for maintaining both Ca2+ and reactive oxygen species homeostasis [23]. Additionally, VDR was found in the prefrontal cortex and parts of the limbic system, and these brain areas had been implicated in the pathophysiology of depression [17].

Another interesting finding in our study was that 21.4% (*P* = 0.048) of older individuals who used antidepressant drugs were classified as having depressive symptoms. Some studies discussed that around 10–30% of individuals with depression presented resistance in drug therapy, not responding to treatment with at least two antidepressants [9, 83–85]. Alternatively, the available antidepressants are commonly accompanied by unpleasant side effects and may cause treatment to be discontinued [33]. Additionally, sometimes unintentional non-adherence, such as forgetting and inability to follow treatment instructions because of poor understanding or physical problems (poor eyesight or impaired manual dexterity), was found to be responsible for treatment discontinuation [86].

In our study we did not perform an evaluation about the influence of antidepressant in 25(OH) D concentration, but some findings point to a relationship in antidepressant use and low level of vitamin D [87, 88]. The mechanism is not well explained, but there are a hypothesis that tricyclic antidepressant may dampen 1-α-hydroxylase activity and induce the activity of 1,25-(OH)2 vitamin D3 24-hydroxylase [87]. If this hypothesis is verified, perhaps this influence of the antidepressant on the level of vitamin D could lead to a risk of depression.

Furthermore, only 9.4% of older adults diagnosed with vitamin D insufficiency or deficiency (39.4 and 25.5%, for insufficiency and deficiency, respectively) were using vitamin D supplements. At the same time, the prevalence of hypovitaminosis (< 30 ng/mL) was present in > 50% of the total sample. These results offer crucial evidence for the lack of vitamin D supplementation in this population.

A growing body of literature has investigated the potential effect of vitamin D on depression and its promising results, such as its prospective role as an adjuvant in drug therapy [89–92]. This issue is still controversial and may be explained by several methodological differences, such as self-reported diagnosis for depression, different vitamin D reference values used, and various methods for serum vitamin D analysis [33, 93–95]. Further studies are needed to confirm these findings, and vitamin D supplementation may become a convenient and low-cost treatment [33, 91]. Moreover, depression is a disease requiring high treatment costs; therefore, there is a substantial gap in the necessity and availability of therapy; consequently, a significant population of depressed individuals are left untreated in low- and middle-income countries [28, 96]. Prevention has the potential to reduce not only recurrences, but also initial episodes, thus reducing the prevalence of period and in a lifetime [97].

Our study presented some limitations. It is essential to mention that the blood tests were performed with a part of the total EpiFloripa Study sample, and this may have limited some of the analyses, such as sex stratification. Clinical and biochemical examinations were conducted at the university, which could select individuals with relatively better health conditions than those from the general population. Although important, the cross-sectional analyzes should be considered with caution due to their methodological limitations inherent in obtaining data. In addition, longitudinal analysis of this data is in progress for a future study.

As for the strengths of this study, we consider that selecting a sample that includes only older adults is one of them because the population is distinct, considering metabolic alterations and life cycle aspects. Aging is a particular physiological phase of the life cycle, in which people reduce their metabolic ability to sustain homeostasis, which renders them highly susceptible to pathological alterations, especially concerning neuropsychiatric disorders [25, 26]. Additionally, we analyzed covariates, such as skin color, ethnicity, and the season in which the blood was collected. The 15-item GDS, which evaluates depressive symptoms, is one of the most widely used screening tools in similar studies and has been validated for application in the Brazilian population. Our study followed a highly accurate method for the quality of the data (trained and supervised interviewers, pilot study, face-to-face interviews, and interview quality control).

## Conclusion

In summary, our findings showed that lower serum 25(OH) D concentrations were independently associated with depressive symptoms in older adults living in Southern Brazil, even after adjusting for demographic, socioeconomic, behavioral, and health variables. These findings are relevant, given the high prevalence of hypovitaminosis D established among this population. We observed that few older adults classified as vitamin D insufficient/deficient were using supplements, pointing to the necessity of serum vitamin D monitoring and prescription, when necessary, for prevention of not only depressive symptoms, but also other well-known conditions, such as compromised bone health. To better elucidate this relationship, a future longitudinal analysis would also be important and should be validated in more detail animal and cell experiments.

## Data Availability

The datasets during and/or analyzed during the current study are available from the corresponding author on reasonable request.

## Abbreviations

25(OH)D: 25-hydroxyvitamin D
BMI: Body mass index
OR: Odds ratio
CI: Confidence interval
MW: Minimum wage
ADLs: Activities of daily living
GDS: Geriatric depression scale
CES-D: Center for epidemiologic studies depression scale
TPH2: tryptophan hydroxylase 2
SERT: serotonin reuptake transporter
MAO-A: monoamine oxidase-A
PDIA3: protein disulfide isomerase family member3
VDR: vitamin D receptors
DLA: daily living activities
NFR2: nuclear factor eritroid-2
g-GT: g-glutamyl transpeptidase
GCLC: glutamate-cysteine ligase
GR: glutathione reductase
Gpx: glutathione peroxidase
ICD‐10: International Statistical Classification of Diseases and Related Health Problems-10 revision
DSM‐IV: Diagnostic and Statistical Manual of Mental Disorders- 5th revision
